# Single nucleotide polymorphisms of nucleotide excision repair pathway are significantly associated with outcomes of platinum-based chemotherapy in lung cancer

**DOI:** 10.1038/s41598-017-08257-7

**Published:** 2017-09-18

**Authors:** Xiao Song, Shiming Wang, Xuan Hong, Xiaoying Li, Xueying Zhao, Cong Huai, Hongyan Chen, Zhiqiang Gao, Ji Qian, Jiucun Wang, Baohui Han, Chunxue Bai, Qiang Li, Junjie Wu, Daru Lu

**Affiliations:** 1 State Key Laboratory of Genetic Engineering and MOE Key Laboratory of Contemporary Anthropology, Institute of Genetics, School of Life Sciences, Fudan University, Shanghai, China; 2 Department of Thoracic Surgery, Shanghai Pulmonary Hospital, Tongji University, Shanghai, China; 3 Department of Thoracic surgery, Shanghai East Hospital, Tongji University School of Medicine, Shanghai, 200120 China; 4 Department of Respiratory Disease, Shanghai Chest Hospital, Shanghai Jiaotong University, Shanghai, China; 5 Department of Pulmonary Medicine, Zhongshan Hospital, Fudan University, Shanghai, China; 6 Department of Pneumology, Changhai Hospital of Shanghai, Second Military Medical University, Shanghai, China

## Abstract

Nucleotide excision repair (NER) pathway plays critical roles in repairing DNA disorders caused by platinum. To comprehensively understand the association between variants of NER and clinical outcomes of platinum-based chemotherapy, 173 SNPs in 27 genes were selected to evaluate association with toxicities and efficiency in 1004 patients with advanced non-small cell lung cancer. The results showed that consecutive significant signals were observed in *XPA, RPA1, POLD1, POLD3*. Further subgroup analysis showed that *GTF2H4* presented consecutive significant signals in clinical benefit among adenocarcimoma. In squamous cell carcinoma, rs4150558, rs2290280, rs8067195 were significantly associated with anemia, rs3786136 was significantly related to thrombocytopenia, *ERCC5* presented consecutive significant signals in response rate. In patients receiving TP regimen, significant association presented in neutropenia, thrombocytopenia and gastrointestinal toxicity. Association with anemia and neutropenia were found in GP regimen. rs4150558 showed significant association with anemia in NP regimen. In patients > 58, *ERCC5* showed consecutive significant signals in gastrointestinal toxicity. Survival analysis showed SNPs in *POLD2*, *XPA*, *ERCC6* and *POLE* were significantly associated with progression free survival, SNPs in *GTF2H4*, *ERCC6*, *GTF2HA*, *MAT1*, *POLD1* were significantly associated with overall survival. This study suggests SNPs in NER pathway could be potential predictors for clinical outcomes of platinum-based chemotherapy among NSCLC.

## Introduction

Lung cancer is one of the most common cancer and the leading cause of cancer-related death worldwide^1^. Despite the improvements of diagnosis and treatment, the prognosis of lung cancer is still poor, and the 5-year-survival rates vary from 4–17% depending on stage and regional differences^2^. Non-small cell lung cancer (NSCLC) accounts for about 80% of primary lung cancer and most patients suffered advanced disease at the time of diagnosis. Two major types of NSCLC are adenocarcimoma (AC) and squamous cell carcinoma (SCC)^3^.

Platinum is an effective antitumor agent and platinum-based chemotherapy is widely used in various cancer treatment^4^. The most commonly used platinum containing agents clinically are cisplatin, carboplatin, oxaliplatin. Cisplatin is first discovered and very commonly used to treat many tumors, including lung cancer^5,6^. The anti-tumor mechanism of platinum compounds is to disorder the DNA replication and induce cell death^7,8^. The most common adduct formed by platinum is intra-strand cross. Cisplatin and carboplatin have the same cross-link, which is 1,2-intrastrand cross links between adjacent purine bases, and oxaliplatin presents a structurally distinct adduct containing a bulky 1,2-diaminocyclohexane group^9^. If the adducts caused by platinum could not be repaired, the disordered DNA could inhibit DNA replication progression, and drive cells into apoptosis^10^.

The damage caused by platinum is recognized and repaired mainly through nucleotide excision repair (NER) pathway^11,12^. DNA damage is repaired by NER via four processes: DNA damage recognition, DNA unwinding, DNA incision, and DNA resynthesis and ligation^10,13^. Many genes involve in these processes. *XPC*, *ERCC6*, and *ERCC8* play important roles in DNA damage recognition, *ERCC2, ERCC3, XPA*, and *RPA1* participate in DNA unwinding, *ERCC1, ERCC4, ERCC5* are responsible for DNA incision^10^. More and more evidences showed that NER was an important mediator of tumor sensitivity to platinum. For example, low expression level of *XPA* and *ERCC1* increased patients′ sensitivity to cisplatin^14,15^, while high level of *ERCC1* was significantly associated with cisplatin resistance. The expression level of *ERCC1* was considered as a potential biomarker for response to cisplatin-based chemotherapy^16,17^. Some studies showed that single nucleotide polymorphisms (SNPs) in NER pathway were also significantly associated with various cancer risk and prognosis, especially the response to platinum-based chemotherapy^18–20^. Some reviews pointed out that there was huge potential clinical value in using mRNA or protein levels of NER genes to predict the response to cisplatin-based chemotherapy for NSCLCs^8,10^, however, the results of studies which investigated the association between SNPs of NER and clinical outcomes of platinum-based treatment are not consistent. In order to fully evaluate the potential clinical value of the SNPs of NER pathway in predicting clinical outcomes of platinum-based chemotherapy for NSCLCs, 1004 Chinese patients with advanced NSCLC who received only platinum-based treatment were enrolled in this study. 173 SNPs located in 27 genes of NER pathway were selected to assess the association between these SNPs and clinical outcomes of platinum-based chemotherapy, including gastrointestinal toxicity, neutropenia, anemia, thrombocytopenia, clinical benefit, response rate, overall survival (OS), and progression-free survival (PFS).

## Results

### Characteristics of patients and clinical outcomes

In order to investigate the association between polymorphisms of NER pathway and clinical outcomes of platinum-based chemotherapy, 1004 patients with advanced NSCLC who received only first-line platinum-based chemotherapy were enrolled in this study. The details of patient characteristics and clinical outcomes were listed in Table 1. The median age of cohort was 58 (ranged from 26 to 82). The patients who were more than 58-year-old accounted for 48.4%, and the ones who were less than or equal to 58-year-old accounted for 51.6%. Most patients were male (70.3%). The percentage of patients with ECOG PS 0–1 was 91.3%. 42.5% of the patients were non-smoker. All patients recruited presented advanced NSCLC, and most of which were stage IV (62.6%). Adenocarcinoma was the most common histological type, which accounted for 57.5%. Platinum-navelbine (NP) (31.5%), platinum-gemcitabine (GP) (23.8%), platinum-paclitaxel (TP) (31.1%), platinum-docetaxel (DP) (8.7%) were the four mainly used chemotherapy regimens in this study. The responses of platinum-based chemotherapy were classified into 4 categories in terms of complete response (CR), partial response (PR), stable disease (SD), and progressive disease (PD) according to Response Evaluation Criteria in Solid Tumors (version 1.0)^21^. Clinical benefit was defined as patients with CR, PR or SD. Response rate contains CR and PR. The response rate was 18.2%, and clinical benefit was 80.7%. The median PFS was 9.1 months and the median OS was 19.3 months. In the toxicity analysis, gastrointestinal toxicity and hematological toxicities including anemia, thrombocytopenia, and neutropenia were collected. 8.3% of patients presented severe gastrointestinal toxicity, 3.1% of patients presented severe anemia, 12.3% of patients presented severe neutropenia, and 3.6% of patients presented severe thrombocytopenia.

**Table 1:** Characteristics and Clinical Outcomes of patient.

**Patient characteristic**	**Total**	**Number**	**%**
Total patient	1004		
Median age(range)	1004	58(26–82)	
Age	1004		
≤58		518	51.6
>58		486	48.4
Gender	1004		
Male		706	70.3
Female		298	29.7
TNM Stage	999		
IIIA		81	8.1
IIIB		293	29.3
IV		625	62.6
ECOG PS	990		
0–1		904	91.3
2		86	8.7
Histological Type	1004		
Adenocarcinoma		632	62.9
Squamous Cell Carcinoma		221	22.0
Adenosquamocarcinoma		20	2.0
Others^a^		131	13.1
Smoking Status^b^	1000		
Never smoker		425	42.5
Ever smoker		575	57.5
Chemotherapy Regimens	1004		
Platinum-navelbine		316	31.5
Platinum-gemcitabine		239	23.8
Platinum-paclitaxel		313	31.1
Platinum-docetaxel		87	8.7
Others platinum combinations		49	4.9
Objective Response	975		
CR		1	0.1
PR		176	18.1
SD		610	60.0
PD		188	19.3
Severe gastrointestinal toxicity	964	80	8.3
Severe hematological toxicity	969		
Anemia	944	29	3.1
Neutropenia	935	115	12.3
Thrombocytopenia	950	34	3.6
Median Time to outcomes (month)	972		
PFS		896	9.1
OS		972	19.3

ECOG PS, Eastern Cooperative Oncology Group performance status; TNM, tumor-node metastasis; CR, complete response; PR, partial response; SD, stable disease; PD, progressive disease; PFS, progression-free survival (months); OS, overall survival (months).

^a^Other carcinomas included mixed cell or undifferentiated carcinoma.

^b^Nonsmokers were defined as those who had smoked <1 cigarette per day and for <1 year in their lifetime.

### Association between the polymorphisms of NER pathway and efficiency of platinum-based chemotherapy

**Figure 1:**
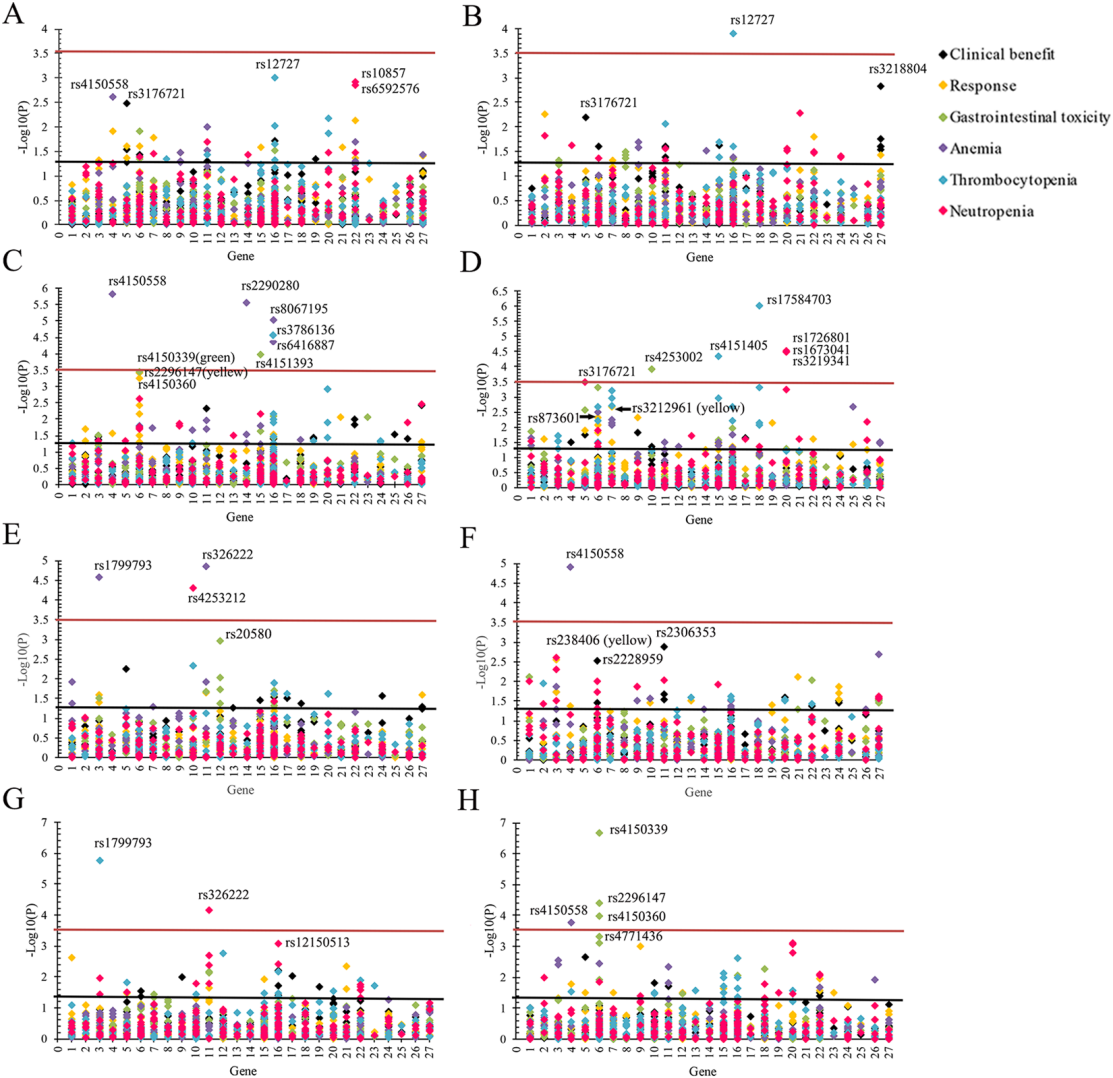
Association analysis between polymorphisms of NER pathway and outcomes of platinum-based chemotherapy in lung cancer. Red line means the significance level after strict Bonferroni correction (*P* < 2.89 × 10^−4^ ((0.05/173)), black line means the significance level of 0.05. (**A**) association analysis in all patients; (**B**) association analysis in subgroup of adenocarcinoma; (**C**) association analysis in subgroup of squamous cell carcinoma; (**D**) association analysis in subgroup of paclitaxel combined with cisplatin regimen; (**E**) association analysis in subgroup of gemcitabine combined with cisplatin regimen; (**F**) association analysis in subgroup of navelbine combined with cisplatin regimen; (**G**) association analysis in subgroup of age ≤ 58; (**H**) association analysis in subgroup of age > 58. The genes analyzed in this study is as follow: 1, *XPC*; 2, *RAD23B*; 3, *ERCC2*; 4, *GTF2H1*; 5, *XPA*; 6, *ERCC5*; 7, *ERCC1*; 8, *ERCC4*; 9, *ERCC8*; 10, *ERCC6*; 11, *DDB2*; 12, *LIG1*; 13, *CDK7*; 14, *CCNH*; 15, *MNAT1*; 16, *RPA1*; 17, *RPA2*; 18, *RFC1*; 19, *RFC2*; 20, *POLD1*; 21, *POLD2*; 22, *POLD3*; 23, *POLD4*; 24, *POLE*; 25, *POLE2*; 26, *GTF2H3*; 27, *GTF2H4*.

To investigate the association between polymorphisms of NER pathway and the efficiency of platinum-based chemotherapy, clinical benefit and response rate were introduced in this study to evaluate the efficacy of platinum-based chemotherapy. There were many polymorphisms presented significant association with clinical benefit and/or response rate of platinum-based chemotherapy (*P* < 0.05), however, after Bonferroni correction, no significant results were remained (*P* < 2.89 × 10^−4^ (0.05/173)) (Fig. 1A). rs3176721 located in *XPA* showed the most significant signal in clinical benefit analysis (χ^2^ test *P* = 0.003; OR = 1.74, 95%CI:1.25–2.44, *P* = 0.001).

Subgroup analyses in different histological types showed that 6 SNPs of *ERCC5* presented consecutive significant signals in response rate in SCC, and rs2296147 showed the most significant result (χ^2^ test *P* = 4.13 × 10^−4^; OR = 0.34, 95%CI:0.20–0.59, *P* = 9.70 × 10^−5^) (Fig. 1C). 4 SNPs located in *GTF2H4* (also known as *P52*) presented consecutive significant signals in clinical benefit in AC and the most significant locus was rs3218804 (χ^2^ test *P* = 0.001; OR = 2.29, 95%CI:1.43–3.66, *P* = 0.001), although no SNPs reached the significant level of Bonferroni correction (Fig. 1B) (Table 2).

**Table 2:** Logistic regression analysis of significant polymorphisms in different groups.

SNP ID	Gene	base change	Group 1^a^			Group 2^b^			OR (95% CI)	*P* value	Clinical outcomes	subgroup
			WT	HE	HO	WT	HE	HO				
rs4150558	*GTF2H1*	T > A	22	6	1	794	96	2	2.74(1.23–6.09)	0.013	anemia	none
rs10857	*POLD3*	A > C	65	46	4	342	370	107	0.55(0.39–0.76)	3.01 × 10^−4^	neutropenia	none
rs6592576	*POLD3*	G > A	65	46	4	346	366	108	0.56(0.41–0.77)	3.58 × 10^−4^	neutropenia	none
rs12727	*RPA1*	C > G	23	7	4	688	205	18	1.81(1.02–3.21)	0.044	thrombocytopenia	none
rs3219281	*POLD1*	G > A	21	10	3	679	222	15	1.87(1.4–3.34)	0.035	thrombocytopenia	none
rs3219341	*POLD1*	G > A	21	10	3	679	220	17	1.84(1.03–3.26)	0.039	thrombocytopenia	none
rs1726801	*POLD1*	G > A	21	10	3	676	213	17	1.86(1.05–3.30)	0.033	thrombocytopenia	none
rs3176721	*XPA*	C > A	92	39	4	385	86	6	1.88(1.28–2.76)	0.001	clinical benefit	AC
rs3218804	*GTF2H4*	G > A	102	32	1	415	62	0	2.29(1.43–3.66)	0.001	clinical benefit	AC
rs4150558	*GTF2H1*	T > A	0	3	0	183	18	1	23.45(2.64–208.13)	0.005	anemia	SCC
rs2290280	*CCNH*	C > A	1	0	2	160	37	8	28.53(1.69–481.13)	0.020	anemia	SCC
rs8067195	*RPA1*	A > G	1	0	2	150	46	9	6.93(1.44–33.49)	0.016	anemia	SCC
rs6416887	*RPA1*	A > G	1	0	2	142	52	10	6.55(1.32–32.44)	0.021	anemia	SCC
rs4150339	*ERCC5*	A > G	9	4	1	165	34	0	3.93(1.15–13.41)	0.029	gastrointestinal toxicity	SCC
rs3786136	*RPA1*	G > A	2	2	1	154	49	1	4.71(1.10–20.12)	0.037	thrombocytopenia	SCC
rs4150339	*ERCC5*	A > G	13	2	2	242	44	2	3.06(1.15–8.19)	0.026	gastrointestinal toxicity	TP
rs4253002	*ERCC6*	G > A	12	5	0	274	13	1	7.81(2.27–26.88)	0.001	gastrointestinal toxicity	TP
rs1726801	*POLD1*	G > A	18	11	4	192	66	2	3.03(1.59–5.77)	0.001	neutropenia	TP
rs1673041	*POLD1*	A > C	7	16	10	134	107	20	3.46(1.97–6.09)	1.70 × 10^−5^	neutropenia	TP
rs3219341	*POLD1*	G > A	18	11	4	193	66	2	3.03(1.59–5.75)	0.001	neutropenia	TP
rs1799793	*ERCC2*	G > A	2	5	0	196	26	3	7.91(2.02–30.96)	0.003	anemia	GP
rs20580	*LIG1*	A > C	2	15	4	110	85	19	3.21(1.53–6.74)	0.002	gastrointestinal toxicity	GP
rs4253212	*ERCC6*	G > A	9	3	2	175	41	1	3.31(1.26–8.72)	0.015	neutropenia	GP
rs4150558	*GTF2H1*	T > A	9	3	1	239	33	0	4.39(1.37–14.08)	0.013	anemia	NP
rs326222	*DDB2*	A > G	23	22	9	251	165	15	2.07(1.32–3.23)	0.001	neutropenia	age ≤ 58
rs12150513	*RPA1*	A > C	31	14	7	263	150	12	2.18(1.32–3.61)	0.002	neutropenia	age ≤ 58
rs4150339	*ERCC5*	A > G	34	5	3	366	61	0	2.53(1.23–5.22)	0.012	gastrointestinal toxicity	age>58
rs2296147	*ERCC5*	A > G	24	12	6	272	146	8	2.10(1.21–3.64)	0.008	gastrointestinal toxicity	age>58
rs4150360	*ERCC5*	G > A	23	13	6	266	152	9	3.07(1.70–5.55)	2.12 × 10^−4^	gastrointestinal toxicity	age>58
rs4771436	*ERCC5*	A > C	31	7	3	190	202	33	0.37(0.19–0.72)	0.003	gastrointestinal toxicity	age>58

AC, Adenocarcinoma; SCC, Squamous Cell Carcinoma; TP, Paclitaxel combined with cisplatin regimen; GP, Gemcitabine combined with cisplatin regimen; NP, navelbine combined with cisplatin regimen; OR, Odd ratio; CI, Confidence interval; WT, wild type; HE, heterozygote; HO, mutant homozygote.

^a^Group 1 means severe toxicity in toxicity analysis, bad response in clinical benefit or response rate analysis.

^b^Group 2 means light toxicity in toxicity analysis, good response in clinical benefit or response rate analysis.

Subgroup analysis among patients receiving different chemotherapy regimens showed that no polymorphisms could achieve the significant level of Bonferroni correction. However, in subgroup of patients receiving NP regimen (Fig. 1F), *ERCC5* and *DDB2* presented consecutive significant signals in clinical benefit, and the most significant signals were rs2228959 (χ^2^ test *P* = 0.003; OR = 2.03, 95%CI:1.04–3.94, *P* = 0.037) in *ERCC5* and rs2306353 (χ^2^ test *P* = 0.001; OR = 0.49, 95%CI:0.29–0.82, *P* = 0.007) in *DDB2*. *ERCC2* showed consecutive significant signals in response rate, and the most significant SNP was rs238406 (χ^2^ test *P* = 0.003; OR = 0.64, 95%CI:0.43–0.95, *P* = 0.025). In subgroup of patients treated with TP regimen (Fig. 1D), *ERCC5* and *ERCC1* showed consecutive significant signals in response rate, and the most significant SNP was rs873601 (χ^2^ test *P* = 0.005; OR = 2.48, 95%CI:1.30–4.75, *P* = 0.006) in *ERCC5*, rs3212961 (χ^2^ test *P* = 0.002; OR = 0.54, 95%CI:0.34–0.86, *P* = 0.009) in *ERCC1* (Table 2). No significant association between polymorphisms of NER pathway and clinical benefit or response rate of platinum-based chemotherapy was found in patients receiving GP regimen (Fig. 1E).

### Association between polymorphisms of NER pathway and the toxicities of platinum-based chemotherapy

Gastrointestinal toxicity and hematological toxicities including anemia, thrombocytopenia, and neutropenia were collected to investigate the association between SNPs of NER pathway and the toxicities of platinum-based chemotherapy. The results showed that *GTF2H1/P62* and *DDB2* presented consecutive significant signals on anemia. *RPA1* and *POLD1* presented consecutive significant signals on thrombocytopenia. *POLD3* presented consecutive significant signals on neutropenia (Fig. 1A). However, no SNPs satisfied the significant level of Bonferroni correction (*P* < 2.89 × 10^−4^).

Subgroup analyses in different histological types showed that rs3786136 in *RPA1* were significantly associated with thrombocytopenia in SCC (χ^2^ test *P* = 3.13 × 10^−5^; OR = 4.71, 95%CI:1.10–20.12, *P* = 0.037) (Fig. 1C) after Bonferroni correction. rs4150558 (χ^2^ test *P* = 1.61 × 10^−6^; OR = 23.45, 95%CI:2.64–208.13, *P* = 0.005) in *GTF2H1*, rs2290280 (χ^2^ test *P* = 2.86 × 10^−6^; OR = 28.53, 95%CI:1.69–481.13, *P* = 0.020) in *CCNH*, rs8067195 (χ^2^ test *P* = 1.01 × 10^−5^; OR = 6.93, 95%CI:1.44–33.49, *P* = 0.016) and rs6416887 (χ^2^ test *P* = 3.07 × 10^−5^; OR = 6.55, 95%CI:1.32–32.44, *P* = 0.021) in *RPA1* were significantly related to anemia in SCC (Fig. 1C) (Table 2).

Subgroup analyses among patients receiving different chemotherapy regimens showed that in subgroup of patients receiving TP regimen (Fig. 1D), rs4253002 in *ERCC6* was significantly associated with gastrointestinal toxicity (χ^2^ test *P* = 1.26 × 10^−4^; OR = 7.81, 95%CI:2.27–26.88, *P* = 0.001). rs4151405 in *MNAT1*(*P* = 4.58 × 10^−5^) and rs17584703 in *RFC1* (*P* = 9.72 × 10^−7^) showed significantly different distribution in thrombocytopenia, however, multiple logistic regression analysis showed that there were no significant association between the 2 SNPs and thrombocytopenia. rs1726801 (χ^2^ test *P* = 3.27 × 10^−5^; OR = 3.03, 95%CI:1.59–5.77, *P* = 0.001), rs1673041 (χ^2^ test *P* = 3.27 × 10^−5^; OR = 3.46, 95%CI:1.97–6.09, *P* = 1.70 × 10^−5^) and rs3219341 (χ^2^ test *P* = 3.09 × 10^−5^; OR = 3.03, 95%CI:1.59–5.75, *P* = 0.001) in *PLOD1* were significantly associated with neutropenia (Table 2). In subgroup of patients receiving GP regimen (Fig. 1E), rs4253212 (χ^2^ test *P* = 4.92 × 10^−5^; OR = 3.31, 95%CI:1.26–8.72, *P* = 0.015) in *ERCC6* was significantly associated with neutropenia. rs1799793 (χ^2^ test *P* = 2.71 × 10^−5^; OR = 7.91, 95%CI:2.02–30.96, *P* = 0.003) in *ERCC2* was significantly associated with anemia. rs20580 (χ^2^ test *P* = 0.001; OR = 3.21, 95%CI:1.53–6.74, *P* = 0.002) in *LIG1* was significantly associated with gastrointestinal toxicity (Table 2). We also found rs4150558 (χ^2^ test *P* = 1.24 × 10^–5^; OR = 4.39, 95%CI:1.37–14.08, *P* = 0.013) in *GTF2H1* were significantly associated with anemia in patients receiving NP regimen (Fig. 1F) (Table 2).

Subgroup analyses in the age of patients ≤ 58 (Fig. 1G) showed that *DDB2* and *RPA1* presented consecutive significant signals on neutropenia. rs326222 (χ^2^ test *P* = 7.43 × 10^−5^; OR = 2.07, 95%CI:1.32–3.23, *P* = 0.001) in *DDB2* remained significant association with neutropenia after Bonferroni correction (Table 2). In the subgroup of patients who were over 58-year-old (Fig. 1H), *ERCC5* showed consecutive significant signals in gastrointestinal toxicity, and 3 SNPs including rs4150339 (χ^2^ test *P* = 2.10 × 10^−7^; OR = 2.53, 95%CI:1.23–5.22, *P* = 0.012), rs2296147 (χ^2^ test *P* = 3.88 × 10^−5^; OR = 2.10, 95%CI:1.21–3.64, *P* = 0.008) and rs4150360 (χ^2^ test *P* = 1.05 × 10^−4^; OR = 3.07, 95%CI:1.70–5.55, *P* = 2.12 × 10^−4^) remained significant association with gastrointestinal toxicity after Bonferroni correction (Table 2).

### Association between polymorphisms of NER and survival of platinum-based chemotherapy

**Figure 2:**
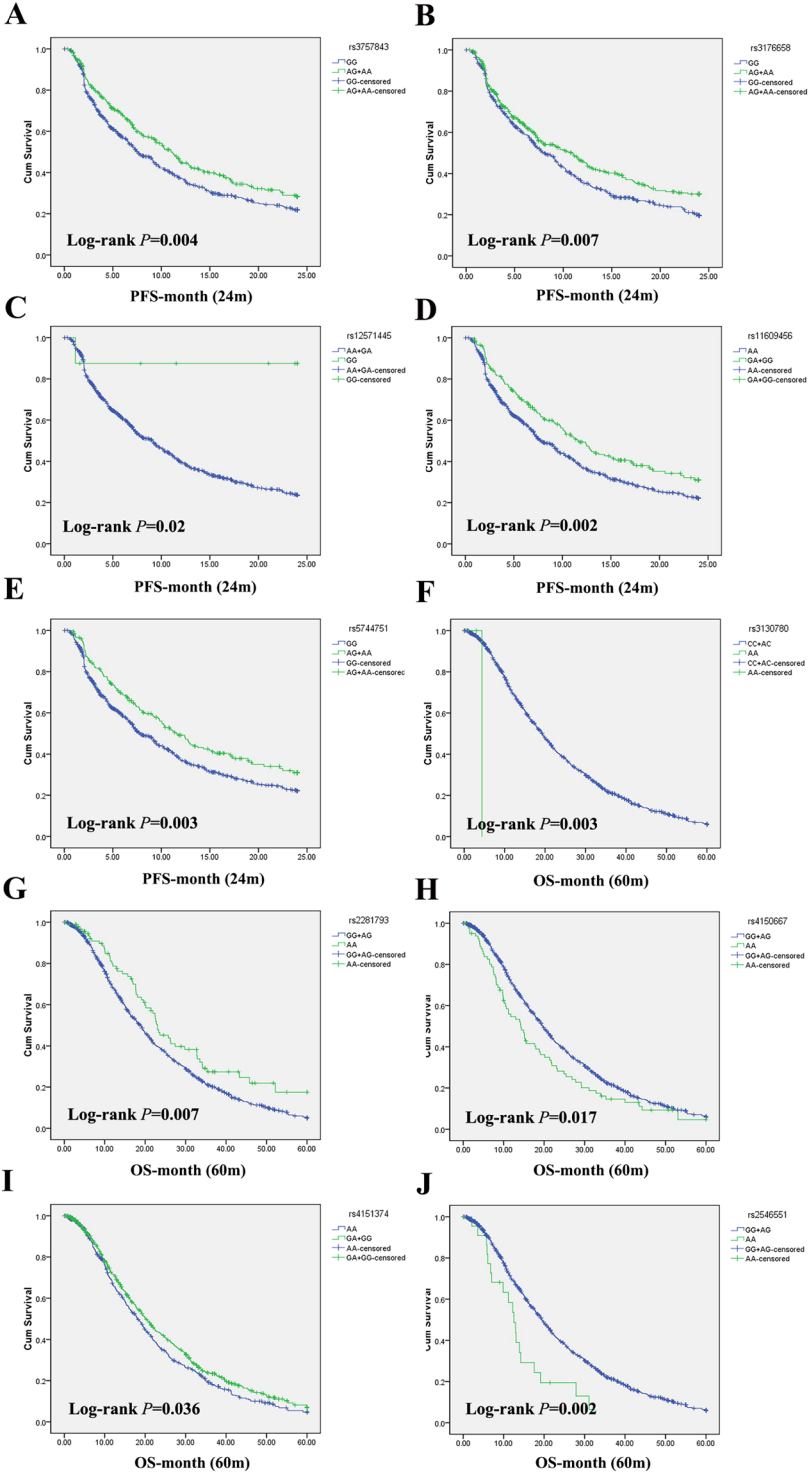
PFS and OS curves of significant polymorphisms of NER pathway. Best models were used in the analysis. (**A**–**E**) showed the results of PFS, and (**F**–**J**) showed the results of OS. (**A**) rs3757843; (**B**) rs3176658; (**C**) rs12571445; (**D**) rs11609456; (**E**) rs5744751; (**F**) rs3130780; (**G**) rs2281793; (**H**) rs4150667; (**I**) rs4151374; (**J**) rs2546551.

Survival analysis was performed to assess the association between the polymorphisms of NER and PFS or OS. The results showed that 5 SNPs were associated with PFS, and all these SNPs decreased the risk of disease progression (Table 3, Fig. 2A–E). rs3757843 (Log-rank *P* = 0.004; HR = 0.78, 95%CI:0.65–0.93, *P* = 0.005) in *POLD2*, rs3176658 (Log-rank *P* = 0.007; HR = 0.81, 95%CI:0.68–0.96, *P* = 0.015) in *XPA*, rs11609456 (Log-rank *P* = 0.002; HR = 0.76, 95%CI:0.62–0.94, *P* = 0.010) and rs5744751 (Log-rank *P* = 0.003; OR = 0.77, 95%CI:0.62–0.94, *P* = 0.011) in *POLE* presented significant association in dominant model. rs12571445 (Log-rank *P* = 0.020; OR = 0.13, 95%CI:0.02–0.93, *P* = 0.042) in *ERCC6* presented significant association when assuming recessive model. In the analysis of OS (Table 4, Fig. 2F–J), rs3130780 (Log-rank *P* = 0.003; HR = 13.65, 95%CI:1.88–99.37, *P* = 0.010) in *GTF2H4*, rs4150667 (Log-rank *P* = 0.017; HR = 1.36, 95%CI:1.06–1.75, *P* = 0.015) in *GTF2H1*, and rs2546551 (Log-rank *P* = 0.002; HR = 1.84, 95%CI:1.15–2.94, *P* = 0.011) in *POLD1* increased the risk of death in recessive model. rs4151374 (Log-rank *P* = 0.036; HR = 0.86, 95%CI:0.75–0.99, *P* = 0.049) in *MAT1* played a significantly protective role in dominant model. rs2281793 (Log-rank *P* = 0.007; HR = 0.70, 95%CI:0.53–0.91, *P* = 0.009) in *ERCC6* could prolong patients’ OS when assuming recessive model.

**Table 3:** Association analysis between polymorphisms of NER and PFS

Gene	SNP ID	Genetic Model^a^	Genotype	MST	Log-rank *P*	Cox proportional hazards regression		
						HR	95%CI	*P*
*POLD2*	rs3757843		G G	7.6	**0.007**	1 (Reference)		
			A G	11.6		0.75	0.63–0.91	**0.003**
			A A	8.7		0.95	0.66–1.37	0.788
		Dom			**0.004**	0.78	0.65–0.93	**0.005**
*XPA*	rs3176658		G G	8.1	**0.027**	1 (Reference)		
			A G	11.0		0.82	0.68–0.98	**0.028**
			A A	10.3		0.77	0.53–1.10	0.149
		Dom			**0.007**	0.81	0.68–0.96	**0.015**
*ERCC6*	rs12571445		A A	9.2	**0.030**	1 (Reference)		
			G A	7.2		1.21	0.97–1.52	0.090
			G G	—		0.13	0.02–0.96	**0.045**
		Rec			**0.020**	0.13	0.02–0.93	**0.042**
*POLE*	rs11609456		A A	7.8	**0.009**	1 (Reference)		
			G A	11.6		0.76	0.62–0.94	**0.011**
			G G	13.7		0.79	0.35–1.78	0.573
		Dom			**0.002**	0.76	0.62–0.94	**0.010**
	rs5744751		G G	7.8	**0.009**	1 (Reference)		
			A G	11.6		0.76	0.62–0.94	**0.011**
			A A	6.7		0.87	0.41–1.85	0.722
		Dom			**0.003**	0.77	0.62–0.94	**0.011**

Add, addictive model; Dom, dominant model; Rec, recessive model; MST, median survival time; HR, hazard ratio; CI, confidence interval; PFS, progression free survival.

^a^The best fitting model was shown.

**Table 4:** Association analysis between polymorphisms of NER and OS

Gene	SNP ID	Genetic Model_a_	Genotype	MST	Log-rank *P*	Cox proportional hazards regression		
						HR	95%CI	*P*
*GTF2H4*	rs3130780		C C	19.5	**0.008**	1 (Reference)		
			A C	17.0		1.06	0.84–1.34	0.639
			A A	4.4		13.71	1.88–99.84	**0.010**
		Rec			**0.003**	13.65	1.88–99.37	**0.010**
*ERCC6*	rs2281793		G G	19.3	**0.018**	1 (Reference)		
			A G	18.3		1.09	0.93–1.27	0.294
			A A	23.0		0.73	0.55–0.96	**0.025**
		Rec			**0.007**	0.70	0.53–0.91	**0.009**
*GTF2H1*	rs4150667		G G	14.2	**0.041**	1 (Reference)		
			A G	19.1		1.02	0.87–1.19	0.815
			A A	20.2		1.37	1.06–1.78	**0.016**
		Rec			**0.017**	1.36	1.06–1.75	**0.015**
*MAT1*	rs4151374		A A	18.1	**0.037**	1 (Reference)		
			G A	21.3		0.84	0.72–0.98	**0.024**
			G G	18.0		0.99	0.77–1.27	0.936
		Dom			**0.036**	0.86	0.75–0.99	**0.049**
*POLD1*	rs2546551		G G	19.0	**0.005**	1 (Reference)		
			A G	21.3		0.91	0.78–1.07	0.250
			A A	12.5		1.79	1.12–2.87	**0.016**
		Rec			**0.002**	1.84	1.15–2.94	**0.011**

Dom, dominant model; Rec, recessive model. MST, median survival time; HR, hazard ratio; CI, confidence interval; OS, overall survival.

^a^The best fitting model was shown.

## Discussion

NER pathway is important in DNA damage repair, especially in repairing the distortion of DNA helical structure^22^. Many genes involved in lesion recognition, DNA unwinding, incision of the DNA around lesion, and finally DNA resynthesis and ligation^13^. Platinum-based chemotherapy is one of the most effective treatments for lung cancer. The mechanism of platinum in cancer treatment is to form intra and inter-strand crosslinks, which could distort the DNA helix, inhibit DNA replication and cause cancer cells apoptosis^5^. NER pathway is the main damage repair system involved in platinum-caused DNA distortion^4^. Many studies focused on the relationship between the expression level of NER-related genes and efficacy of platinum-based treatment for cancer. The status of *ERCC1* protein expression was reported as a predictive marker for outcomes of platinum-based chemotherapy in lung cancer^17^. Some studies also pointed out those SNPs in some members of NER pathway showed significant association with clinical outcomes of platinum-based chemotherapy. The polymorphisms of *XPD* were significantly associated with not only efficiency but also severe toxicity of platinum-based chemotherapy in lung cancer^23,24^. Other members of NER pathway, such as *XPA, ERCC5*, and *ERCC2*, were related to the response of platinum-based chemotherapy in lung cancer^15,25,26^. In order to comprehensively assess the association between polymorphisms of NER pathway and clinical outcomes of platinum-based chemotherapy, a total of 173 SNPs located in 27 genes were investigated in this study to evaluate their association with gastrointestinal toxicity, neutropenia, anemia, thrombocytopenia, clinical benefit, response rate, overall survival (OS), and progression-free survival (PFS).

Our results showed that variants in NER pathway were significantly associated with clinical outcomes of platinum-based chemotherapy. Polymorphisms in *XPA*, *DDB2* and *GTF2H4* were significantly associated with clinical benefit. Polymorphisms in *ERCC2*, *ERCC5* were significantly associated with response rate. Polymorphisms in *GTF2H1*, *ERCC2* and *RPA1* showed significant association with anemia. Polymorphisms in *RPA1* showed significant association with thrombocytopenia. Polymorphisms in *ERCC2*, *ERCC6*, *DDB2*, *RPA1*, *POLD1* and *POLD3* presented significant association with neutropenia. Polymorphisms in *POLD2*, *XPA*, *ERCC6*, *POLE* presented significant association with PFS. Polymorphisms in *GTF2H4*, *ERCC6*, *GTF2H1*, *MAT1* and *POLD1* presented significant association with OS.

*XPA* encodes a zinc-finger DNA-binding protein, and plays an important role of damage recognition in NER pathway^27^. Genetic variants in *XPA* were significantly associated with lung cancer risk^28^. Knockdown the expression of *XPA* could sensitize NSCLC-derived cell lines to cisplatin^29^. Our results showed that rs3176721 in *XPA* was significantly associated with clinical benefit in all patients, as well as in AC subgroup. rs3176658 in *XPA* was significantly associated with PFS, and the A allele could significantly decrease the risk of disease progression.

*DDB2* is a component of *DDB* which is the damage-specific DNA-binding heterodimeric complex^30^. SNPs in *DDB2* were significantly associated with the risk of lung cancer^31^. A recent GWAS analysis showed that rs747650 in DDB2 was a new susceptibility locus of severe acne^32^. Overexpression of *DDB2* could sensitize the cancer cells to cisplatin treatment which indicated that DDB2 may play important role in platinum-based chemotherapy^33^. In our study, we found that rs2306353 significantly associated with clinical benefit in patients receiving NP regimen, and rs326222 in *DDB2* were significantly risk factor for neutropenia in subgroup of patients younger than 58 years old.

*GTF2H4* (also known as P52) encodes a subunit of transcription factor II H (TFIIH), and is known to be involved in nucleotide excision repair^34^. In a recent study of a large-scale analysis of six published GWAS datasets pointed out that rs114596632 in *GTF2H4* was significantly associated with lung cancer risk^35^, rs2074508 in *GTF2H4* was significantly associated with smoking-related lung cancer^36^. In the current study, *GTF2H4* presented consecutive significant signals in clinical benefit among AC patients. rs3130780 in *GTF2H4* was significantly associated with OS, and AA genotype could significantly increase risk of death.

*ERCC5* plays important roles in DNA incision in NER pathway. *ERCC5* is a well-known gene which has great impact on cancer. Our study showed that *ERCC5* presented consecutive significant signals not only in response rate in SCC, but also in gastrointestinal toxicity among patients > 58 years old. rs2296147 was the most significant SNP which associated with response rate. It was reported that rs2296147 was not only associated with cancer risk, but also related to prognosis of cancer^37^. There were also many studies showed that rs2296147 was associated with prognosis of advanced non-small cell lung cancer treated with platinum-based chemotherapy, and could predict the clinical outcomes of platinum-based chemotherapy^38–41^. rs2296147 is located in the promoter of *ERCC5*. The transcription repressor of *SNAI1* is predicted to bind to the sequence around rs2296147, which indicating that rs2296147 may take part in negative regulating the expression of *ERCC5*.

*RPA1* is an important subunit of *RPA* which is a major eukaryotic single-strand DNA-binding protein complex, and essential for DNA repair, DNA replication, DNA recombination, telomere maintenance, activation of DNA damage checkpoints and the maintenance of genomic integrity^42^. *RPA1* is also reported as a part of the replication fork protection complex^43^. Previous studies showed that *RPA1* played important roles in Pt-DNA repair^44^, and expression level of *RPA1* could be used to predict prognosis of cancer^45^. However, no studies focused on the relationship between RPA1 and the hematological toxicities of platinum-based chemotherapy. In this study, we found that polymorphisms in *RPA1* presented significant association with all 3 hematological toxicities. rs12727 and rs3786136 showed significant association with thrombocytopenia, rs8067195 and rs6416887 showed significant association with anemia, rs12150513 showed significant association with neutropenia. rs12727 is located in the 3′UTR of *RPA1*, and the sequence around it is the potential target of miR-345-3p, miR-6732-3p and miR-6771-3p. *RPA1* is also a target of PTEN function in fork protection to maintain genome stability^46^.

*ERCC6* can recognize DNA damage and recruit NER repair factors to the DNA damage site. Polymorphisms in *ERCC6* showed significant association with the risk and prognosis of lung cancer^47^. Previous study showed that no statistically significant association was found between the platinum-related toxicities and SNPs of *ERCC6* or*, CCNH* ^48^. In our study, we found that rs4253002 in *ERCC6* showed significant association with gastrointestinal toxicity in the patients receiving TP regimen, and rs4253212 in *ERCC6* showed significant association with neutropenia in the patients receiving GP regimen. We also found rs2290280 in *CCNH* was significantly associated with anemia in SCC subgroup. In survival analysis, rs12571445 in *ERCC6* showed significant association with PFS, and rs2281793 in *ERCC6* showed significant association with OS. Our results suggested that both *ERCC6* and *CCNH* might involve in regulating clinical outcomes of platinum-based chemotherapy.

DNA polymerase δ is conserved from humans to yeast, and performs important functions in DNA replication and repair processes. The Polδ complex was comprised of four subunits (p125, p66, p50 and p12) which encoded by *POLD1, POLD3*, *POLD2 and POLD4* ^49^. Polymorphisms and mutations in *POLD1* and *POLD3* were reported to be associated with cancer risk^50,51^. Overexpression of *POLD1* was associated with platinum resistance in a long-term survivor of mesothelioma^52^. In this study, *POLD1* and *POLD3* showed significant association with neutropenia. rs1726801, rs1673041 and rs3219341 in *POLD1* showed significant association with neutropenia in patients receiving TP regimen. rs10857 and rs6592576 in *POLD3* showed significant association with neutropenia in all patients. rs3757843 in *POLD2* showed significant association with PFS, and rs2546551 in *POLD1* showed significant association with OS.

We also found that rs11609456 and rs5744751 in *POLE* showed significant association with PFS, rs4151374 in *MAT1* and rs4150667 in *GTF2H1* showed significant association with OS. rs4150558 in *GTF2H1* was significantly associated with anemia in all patients, the same effect was also observed in not only SCC but also subgroup of patients receiving NP regimen. Our results showed that some of the significant signals of χ^2^ test were absent in multiple logistic regression analysis, especially in subgroup analysis. For example, rs12727 in *RPA1* showed in significantly different distribution in thrombocytopenia in AC subgroup, rs4151405 in *MNAT1* and rs17584703 in *RFC1* showed significantly different distribution in thrombocytopenia in patients receiving TP regimen, however, multiple logistic regression analysis showed no significant association. This might be because that the number of patients were few in some subgroups, resulting in the distribution of genotypes disequilibrium and significant signals of χ^2^ test. However, *P* value for trend as well as OR and 95%CI were used in multiple logistic regression analysis, which reveal the real relationship or association between clinical outcomes and polymorphisms.

In the current study, subgroups analysis of chemotherapy regimen was carried out to investigate other drugs affect the results of association analysis of platinum. We found that different genes were associated with different outcomes in different subgroups, which suggested that other drugs effect might have impact on clinical outcomes of platinum-based treatment and subgroup analysis was important in platinum-related pharmacogenetics studies. In survival analysis, some significant signals were only presented in heterozygote, but disappeared in mutant homozygote. This phenomenon was termed “heterozygote advantage”. Many other studies showed the similar results. For example, there was a clear association between heterozygosity at the *TIRAP* S180L locus and protection against multiple infectious diseases^53^. In breast cancer that the heterozygous genotype of 5′ UTR -26 G > A polymorphism located in *BRCA2* was found to be protective effect in cancer risk. Our results also showed that heterozygous genotype was significantly associated with good prognosis^54^. In some subgroups of survival analysis, especially in recessive model, such as rs12571445 (*ERCC6*) in PFS analysis, and rs3130780 (*GTF2H4*) and rs2546551 (*POLD1*) in OS analysis, the sample size of homozygous mutation is too small to get reliable results, and more samples are needed to confirm the results.

Summary, 173 SNPs located in 27 genes of NER pathway were investigated in this study to assess the association with clinical outcomes of platinum-based chemotherapy for advanced NSCLC. SNPs in *ERCC2* (rs1799793)*, ERCC5* (rs4150339, rs2296147, rs4150360, rs4771436)*, ERCC6* (rs4253002, rs4253212, rs12571445, rs2281793)*, XPA* (rs3176721, rs3176658)*, GTF2H1* (rs4150558, rs4150667)*, GTF2H4* (rs3218804, rs3130780)*, DDB2* (rs326222)*, RPA1* (rs12727, rs8067195, rs6416887, rs3786136, rs12150513)*, POLD1* (rs3219281, rs3219341, rs1726801, rs1673041, rs2546551)*, POLD2* (rs3757843)*, POLD3* (rs10857, rs6592576)*, POLE* (rs11609456, rs5744751) and *MAT1* (rs4151374) showed significant association with toxicities and efficiency of platinum-based chemotherapy in different subgroups. Due to the low incidence of severe toxicity, statistics power is not sufficient in some groups, validation assay and functional investigation is needed in future study.

## Methods

### Study population

1004 patients recruited in current study were histopathologically diagnosed stage IIIA-IV NSCLC patients in Shanghai, China. Each patient was informed consent before enrolled. The criteria for recruitment were defined as below: (1) the patients enrolled in this study was over 18 years old; (2) the patients were newly diagnosed, and only received platinum-based chemotherapy. Any patient with surgery, radiotherapy, concurrent chemoradiotherapy or previous chemotherapy was excluded; (3) the performance status was between 0 and 2; (4) there were no other malignancy in the past 5 years; (5) no cardiac arrhythmias, no active congestive heart failure, and no uncontrolled clinical infections; (6) the absolute neutrophil count ≥ 1.5 × 10^9^ cells/L, platelets ≥ 100 × 10^9^cells/L, creatinine clearance ≥ 60 mL/min, serum creatinine ≤ 1.5 × upper limit normal, alanine and aspartate aminotransferase ≤ 1.5 × upper limit normal. All the methods mentioned in the protocol were carried out in accordance with the institutional guidelines and approved by the Ethical Review Committee of Fudan University, and informed consent was obtained from all patients before samples collection.

Clinical outcomes including toxicities, responses and survival were evaluated in the current study. The responses to platinum-based chemotherapy were assessed after two cycles of treatment, and the responses were classified into 4 categories in terms of complete response (CR), partial response (PR), stable disease (SD), and progressive disease (PD) according to response evaluation criteria in solid tumors (version 1.0)^21^. Clinical benefit was defined as patients with CR, PR or SD. Response rate contains CR and PR. Gastrointestinal toxicity and hematologic toxicities including neutropenia, anemia, and thrombocytopenia, were collected and evaluated twice a week according to the Common Terminology Criteria for Adverse Events V3.0 (CTCAE 3.0). Grade 3 or 4 toxicities were defined as severe adverse effects. Grade 5 toxicity, also known as death, was not observed in this study. Progression-free survival (PFS) and overall survival (OS) were assessed in the survival analysis. PFS was calculated from the date of first cycle of platinum-based chemotherapy to the date of PD, death, or the last follow-up. OS was calculated from the date of first cycle of platinum-based chemotherapy to the date of death or the last follow-up. The survival data was collected from follow-up calls, and the Social Security Death Index and inpatient and outpatient clinical medical records.

### SNPs selection and genotyping

Base on the genotype data of Han Chinese in Beijing (CHB) from phase II Hapmap SNP database, 173 SNPs of 27 genes involved in NER pathway were selected using the strategies of tag-SNPs and functional SNPs by Haplowview 4.1 (http://www.broadinstitute.org/haploview) with the criteria of minor allele frequency ≥ 0.05 and correlation coefficient ≥ 0.8. The detail information was listed in Supplementary Table 1.

Human genomic DNA was extracted from blood samples using Qiagen Blood Kit (Qiagen, CA). All SNPs were genotyped using iSelect HD BeadChip (Illumina, San Diego, Calif). The results of random duplicate assays were consistent. Following the criteria of SNP genotyping call rate > 0.95, MAF > 0.01, GenCall score > 0.2, all 173 SNPs located in 27 genes (detailed in supplementary Table 1) were included in final analysis.

### Statistical analysis

Demographic and clinical factors were test against clinical outcomes by chi-square tests or log-rank test. Factors that had *P*-value < 0.05 were regarded as covariates (Supplementary Table 2, Supplementary Table 3). The Chi-square test was used to assess whether SNPs’ genotypes were significantly different in the distribution of clinical outcomes. Bonferroni correction was performed by multiplying the number of all SNPs tested in the study to control for multiple comparisons. Significant SNPs from Chi-square were included in multiple logistic regression adjusted for covariates to estimate their association with clinical outcomes by odds ratio (OR) and confidence interval (CI). Log-rank test was used to compare the survival curve between patients’ groups. Cox proportional hazards regression adjusted for covariates was performed to evaluate the association between survival and significant polymorphisms SNPs from log-rank test by hazard ratios (HRs) with 95% CIs in additive, dominant, or recessive model. All *P*-values presented were two-sided, and a level of *P* < 0.05 was considered statistically significant. SPSS software (SPSS, Chicago, IL) and PLINK v1.07 were used for statistical analyses in this study.

## Electronic supplementary material

Supplementary information
