# Supplementary material for: Single nucleotide polymorphisms of nucleotide excision repair pathway are significantly associated with outcomes of platinum-based chemotherapy in lung cancer
Source: Sci Rep. 2017 Sep 18;7:11785. doi: 10.1038/s41598-017-08257-7 (PMC5603542; doi:10.1038/s41598-017-08257-7)
Supplement: Supplementary file 1 — Supplementary information [file 41598_2017_8257_MOESM1_ESM.pdf]

Single nucleotide polymorphisms of nucleotide excision repair pathway are significantly associated with outcomes of platinum-based chemotherapy in lung cancer

Xiao Song[1,2#], Shiming Wang[1#], Xuan Hong[3#], Xiaoying Li[1], Xueying Zhao[1], Cong Huai[1], Hongyan Chen[1], Zhiqiang Gao[4], Ji Qian[1], Jiucun Wang[1], Baohui Han[4], Chunxue Bai[5], Qiang Li[6], Junjie Wu[1], Daru Lu[1*]

Gene	NCBI SNP ID	Position[a]	Functional Consequence	Base Change	HWE (*P*-value)	Genotyping rate (%)	MAF	Genotype frequency		
								Wild Type	Heterozygote	Mutant Type
RPA2	rs7356	chr1:27891589	3'UTR	G:A	0.992	99.8	0.450	304 (30.3%)	495 (49.4%)	203 (20.3%)
	rs17257252	chr1:27893973	intron	C:A	0.214	100.0	0.125	763 (76.0%)	230 (22.9%)	11 (1.1%)
XPC	rs2228001	chr3:14145949	missense	A:C	0.819	97.5	0.354	410 (41.9%)	444 (45.4%)	125 (12.8%)
	rs2733533	chr3:14147200	intron	C:A	1.000	100.0	0.048	909 (90.5%)	93 (9.3%)	2 (0.2%)
	rs2607737	chr3:14151668	intron	G:A	0.888	100.0	0.295	497 (49.5%)	421 (41.9%)	86 (8.6%)
	rs3731114	chr3:14165122	intron	G:C	0.078	94.8	0.202	616 (64.7%)	288 (30.3%)	48 (5.0%)
	rs3731054	chr3:14179017	5'flanking	C:G	0.930	99.8	0.336	443 (44.2%)	445 (44.4%)	114 (11.4%)
RFC1	rs1057807	chr4:39287853	3'UTR	A:G	0.717	100.0	0.363	411 (40.9%)	458 (45.6%)	135 (13.4%)
	rs13147094	chr4:39309262	intron	G:A	1.000	100.0	0.193	654 (65.1%)	313 (31.2%)	37 (3.7%)
	rs7658917	chr4:39332767	intron	G:A	1.000	100.0	0.414	345 (34.4%)	487 (48.5%)	172 (17.1%)
	rs3796517	chr4:39335333	intron	A:G	0.733	100.0	0.164	699 (69.6%)	280 (27.9%)	25 (2.5%)
	rs2306596	chr4:39342320	intron	A:C	1.000	99.9	0.358	413 (41.2%)	462 (46.1%)	128 (12.8%)
	rs17584703	chr4:39363236	intron	A:G	0.792	100.0	0.256	557 (55.5%)	379 (37.7%)	68 (6.8%)
	rs6851075	chr4:39366034	intron	A:G	0.829	99.9	0.172	689 (68.7%)	283 (28.2%)	31 (3.1%)
	rs3736168	chr4:39366463	5'flanking	G:A	0.633	99.9	0.428	332 (33.1%)	483 (48.2%)	188 (18.7%)
ERCC8	rs3117	chr5:60874510	3'UTR	A:G	1.000	99.9	0.070	867 (86.4%)	131 (13.1%)	5 (0.5%)
	rs4647150	chr5:60874778	intron	G:A	0.405	99.9	0.171	685 (68.3%)	293 (29.2%)	25 (2.5%)
	rs4647130	chr5:60887482	synonymous	A:G	0.022	99.8	0.072	868 (86.6%)	123 (12.3%)	11 (1.1%)
	rs12657309	chr5:60910298	intron	A:G	0.273	99.8	0.220	603 (60.2%)	357 (35.6%)	42 (4.2%)
	rs976080	chr5:60921824	intron	C:A	0.951	100.0	0.067	874 (87.1%)	125 (12.5%)	5 (0.5%)

	rs158922	chr5:60945159	5'UTR	G:A	1.000	100.0	0.070	868 (86.5%)	131 (13.0%)	5 (0.5%)
	rs158920	chr5:60945441	5'UTR	G:A	0.537	99.9	0.099	816 (81.4%)	175 (17.4%)	12 (1.2%)
CDK7	rs2972363	chr5:69257549	intron	G:A	1.000	100.0	0.177	680 (67.7%)	293 (29.2%)	31 (3.1%)
	rs4423955	chr5:69272148	intron	G:A	0.233	99.8	0.424	323 (32.2%)	509 (50.8%)	170 (17.0%)
	rs6865178	chr5:69274130	intron	G:A	0.264	100.0	0.442	303 (30.2%)	514 (51.2%)	187 (18.6%)
CCNH	rs2230641	chr5:87399457	missense	A:G	0.027	100.0	0.102	803 (80.0%)	197 (19.6%)	4 (0.4%)
	rs3093816	chr5:87401570	intron	G:A	0.673	100.0	0.479	269 (26.8%)	509 (50.7%)	226 (22.5%)
	rs2290280	chr5:87412904	5'UTR	C:A	0.004	100.0	0.135	762 (75.9%)	212 (21.1%)	30 (3.0%)
	rs1807895	chr5:87413020	5'UTR	A:C	0.619	99.6	0.355	420 (42.0%)	450 (45.0%)	130 (13.0%)
GTF2H4	rs3130780	chr6:30906531	5'flanking	C:A	1.000	100.0	0.054	899 (89.5%)	102 (10.2%)	3 (0.3%)
	rs2284175	chr6:30907368	5'flanking	G:A	0.568	100.0	0.409	355 (35.4%)	476 (47.4%)	173 (17.2%)
	rs3218803	chr6:30907503	5'flanking	A:G	0.568	100.0	0.409	355 (35.4%)	476 (47.4%)	173 (17.2%)
	rs3218804	chr6:30907826	5'flanking	G:A	0.314	100.0	0.076	855 (85.2%)	146 (14.5%)	3 (0.3%)
	rs2284176	chr6:30907845	5'flanking	G:A	0.606	99.5	0.411	351 (35.1%)	475 (47.5%)	173 (17.3%)
	rs3218806	chr6:30907887	5'flanking	A:C	0.677	99.4	0.411	350 (35.1%)	476 (47.7%)	172 (17.2%)
	rs1264309	chr6:30908122	5'flanking	A:G	0.520	99.4	0.264	536 (53.7%)	397 (39.8%)	65 (6.5%)
	rs2074510	chr6:30908257	5'UTR	A:C	0.568	100.0	0.409	355 (35.4%)	476 (47.4%)	173 (17.2%)
	rs1052693	chr6:30908375	5'UTR	A:G	0.546	99.9	0.409	355 (35.4%)	475 (47.4%)	173 (17.2%)
	rs3218822	chr6:30912559	intron	A:G	0.535	100.0	0.066	877 (87.4%)	121 (12.1%)	6 (0.6%)
POLD2	rs3087363	chr7:44118068	intron	G:A	0.119	100.0	0.077	860 (85.7%)	134 (13.3%)	10 (1.0%)
	rs3087370	chr7:44122210	5'UTR	A:G	0.588	100.0	0.236	583 (58.1%)	369 (36.8%)	52 (5.2%)
	rs2979422	chr7:44122756	intron	A:G	0.233	100.0	0.203	644 (64.1%)	312 (31.1%)	48 (4.8%)
	rs3757843	chr7:44123958	5'flanking	G:A	0.873	99.8	0.216	617 (61.6%)	337 (33.6%)	48 (4.8%)
RFC2	rs3135712	chr7:74232049	3'UTR	G:A	0.767	100.0	0.089	835 (83.2%)	160 (15.9%)	9 (0.9%)
	rs3135695	chr7:74236235	intron	C:A	0.867	99.9	0.115	785 (78.3%)	206 (20.5%)	12 (1.2%)

	rs3135670	chr7:74246577	intron	A:C	1.000	94.2	0.084	794 (83.9%)	146 (15.4%)	6 (0.6%)
XPA	rs3176721	chr9:97679912	intron	C:A	1.000	100.0	0.112	792 (78.9%)	200 (19.9%)	12 (1.2%)
	rs3176690	chr9:97685195	intron	A:C	1.000	100.0	0.081	847 (84.4%)	151 (15.0%)	6 (0.6%)
	rs3176689	chr9:97685514	intron	A:T	0.387	98.7	0.356	418 (42.2%)	441 (44.5%)	132 (13.3%)
	rs3176658	chr9:97691581	intron	G:A	0.815	99.2	0.248	565 (56.7%)	368 (36.9%)	63 (6.3%)
	rs1800975	chr9:96797296	5'UTR	A:G	0.763	99.8	0.498	250 (25.0%)	507 (50.6%)	245 (24.5%)
RAD23B	rs7041137	chr9:107282291	5'flanking	G:A	0.184	100.0	0.211	633 (63.0%)	319 (31.8%)	52 (5.2%)
	rs17195974	chr9:107285391	intron	A:G	1.000	99.9	0.215	618 (61.6%)	339 (33.8%)	46 (4.6%)
	rs7031906	chr9:107287196	intron	C:A	0.597	95.5	0.249	544 (56.7%)	352 (36.7%)	63 (6.6%)
	rs10816484	chr9:107289586	intron	A:G	1.000	100.0	0.376	391 (38.9%)	471 (46.9%)	142 (14.1%)
ERCC6	rs11101137	chr10:49456985	3'UTR	A:G	0.939	100.0	0.390	374 (37.3%)	476 (47.4%)	154 (15.3%)
	rs3750751	chr10:49457882	3'UTR	G:A	0.218	100.0	0.167	691 (68.8%)	291 (29.0%)	22 (2.2%)
	rs4253231	chr10:49458762	3'UTR	A:G	0.192	100.0	0.168	689 (68.6%)	293 (29.2%)	22 (2.2%)
	rs4838519	chr10:49462211	intron	A:C	0.777	99.9	0.489	265 (26.4%)	496 (49.5%)	242 (24.1%)
	rs4253212	chr10:49470166	intron	G:A	0.850	99.9	0.110	796 (79.4%)	194 (19.3%)	13 (1.3%)
	rs2228526	chr10:49470671	missense	A:G	0.906	100.0	0.053	899 (89.5%)	103 (10.3%)	2 (0.2%)
	rs12571445	chr10:49514137	intron	A:G	0.880	100.0	0.085	841 (83.8%)	155 (15.4%)	8 (0.8%)
	rs2281793	chr10:49519496	intron	G:A	0.417	99.7	0.323	453 (45.3%)	450 (45.0%)	98 (9.8%)
	rs2228528	chr10:49524234	missense	G:A	0.575	100.0	0.459	299 (29.8%)	489 (48.7%)	216 (21.5%)
	rs4253002	chr10:49539292	5'flanking	G:A	0.301	100.0	0.046	916 (91.2%)	84 (8.4%)	4 (0.4%)
GTF2H1	rs3802968	chr11:18322331	5'flanking	G:A	0.875	100.0	0.024	957 (95.3%)	46 (4.6%)	1 (0.1%)
	rs4150558	chr11:18332808	intron	T:A	0.978	97.6	0.061	865 (88.3%)	111 (11.3%)	4 (0.4%)
	rs4150628	chr11:18344321	intron	A:G	0.296	100.0	0.135	746 (74.3%)	244 (24.3%)	14 (1.4%)
	rs4150667	chr11:18361364	intron	G:A	0.387	100.0	0.298	488 (48.6%)	433 (43.1%)	83 (8.3%)
DDB2	rs12578030	chr11:47213050	5'flanking	A:G	0.841	100.0	0.044	917 (91.3%)	86 (8.6%)	1 (0.1%)

	rs2029298	chr11:47213167	5'flanking	G:A	0.356	100.0	0.302	496 (49.4%)	410 (40.8%)	98 (9.8%)
	rs11039138	chr11:47227672	intron	G:A	0.315	100.0	0.027	952 (94.8%)	50 (5.0%)	2 (0.2%)
	rs2306353	chr11:47235157	intron	A:G	0.806	99.9	0.328	455 (45.4%)	438 (43.7%)	110 (11.0%)
	rs4647756	chr11:47237428	intron	A:C	0.932	100.0	0.053	900 (89.6%)	102 (10.2%)	2 (0.2%)
	rs326222	chr11:47238117	intron	A:G	0.720	100.0	0.244	577 (57.5%)	365 (36.4%)	62 (6.2%)
POLD4	rs1790735	chr11:67355012	5'flanking	A:G	0.065	100.0	0.139	752 (74.9%)	225 (22.4%)	27 (2.7%)
POLD3	rs10899013	chr11:74598497	intron	G:A	0.774	100.0	0.234	591 (58.9%)	356 (35.5%)	57 (5.7%)
	rs7940880	chr11:74606369	intron	G:A	0.308	100.0	0.370	407 (40.5%)	452 (45.0%)	145 (14.4%)
	rs6592576	chr11:74615776	intron	G:A	0.626	100.0	0.340	441 (43.9%)	443 (44.1%)	120 (12.0%)
	rs6592577	chr11:74616569	intron	A:G	0.964	100.0	0.291	504 (50.2%)	416 (41.4%)	84 (8.4%)
	rs12807883	chr11:74616947	intron	A:G	0.777	100.0	0.056	895 (89.1%)	105 (10.5%)	4 (0.4%)
	rs10857	chr11:74642326	3'UTR	A:C	0.814	99.9	0.341	437 (43.6%)	447 (44.6%)	119 (11.9%)
	rs1051076	chr11:74642554	3'UTR	A:G	1.000	100.0	0.040	924 (92.0%)	79 (7.9%)	1 (0.1%)
GTF2H3	rs10773020	chr12:123632542	5'flanking	A:C	1.000	100.0	0.087	836 (83.3%)	161 (16.0%)	7 (0.7%)
	rs10846535	chr12:123645841	intron	A:G	0.769	99.8	0.150	726 (72.5%)	252 (25.1%)	24 (2.4%)
	rs11057318	chr12:123655941	intron	G:A	0.021	100.0	0.056	899 (89.5%)	97 (9.7%)	8 (0.8%)
	rs6488888	chr12:123658044	intron	A:G	0.443	100.0	0.063	879 (87.5%)	123 (12.3%)	2 (0.2%)
POLE	rs14302	chr12:132624162	3'UTR	G:A	0.835	99.9	0.208	630 (62.8%)	328 (32.7%)	45 (4.5%)
	rs4883543	chr12:132625418	intron	A:G	0.458	99.9	0.320	458 (45.7%)	448 (44.7%)	97 (9.7%)
	rs4883624	chr12:132636151	intron	A:G	0.270	100.0	0.322	453 (45.1%)	455 (45.3%)	96 (9.6%)
	rs11146986	chr12:132641691	synonymous	G:A	0.240	100.0	0.093	823 (82.0%)	176 (17.5%)	5 (0.5%)
	rs11609456	chr12:132654462	intron	A:G	0.546	99.9	0.112	789 (78.7%)	204 (20.3%)	10 (1.0%)
	rs5744751	chr12:132677409	missense	G:A	0.733	100.0	0.113	789 (78.6%)	204 (20.3%)	11 (1.1%)
ERCC5	rs2094258	chr13:102844409	5'flanking	G:A	0.681	100.0	0.387	373 (37.2%)	484 (48.2%)	147 (14.6%)
	rs751402	chr13:102845848	5'UTR	G:A	0.845	99.5	0.335	440 (44.0%)	449 (44.9%)	110 (11.0%)

	rs2296147	chr13:102846025	5'UTR	A:G	0.875	99.9	0.211	625 (62.3%)	332 (33.1%)	46 (4.6%)
	rs16960632	chr13:102847365	intron	A:G	0.116	100.0	0.143	731 (72.8%)	259 (25.8%)	14 (1.4%)
	rs4771436	chr13:102849670	intron	A:C	0.506	99.3	0.295	490 (49.1%)	425 (42.6%)	82 (8.2%)
	rs1047768	chr13:102852167	synonymous	A:G	0.475	99.9	0.279	516 (51.4%)	414 (41.3%)	73 (7.3%)
	rs2228959	chr13:102865812	synonymous	C:A	0.166	100.0	0.074	865 (86.2%)	130 (12.9%)	9 (0.9%)
	rs4150339	chr13:102867901	intron	A:G	0.159	100.0	0.084	847 (84.4%)	146 (14.5%)	11 (1.1%)
	rs4150360	chr13:102872412	intron	G:A	0.674	100.0	0.213	625 (62.3%)	331 (33.0%)	48 (4.8%)
	rs8002276	chr13:102873789	intron	A:G	0.780	99.9	0.426	328 (32.7%)	496 (49.5%)	179 (17.8%)
	rs4150383	chr13:102874880	intron	G:A	0.375	100.0	0.048	912 (90.8%)	88 (8.8%)	4 (0.4%)
	rs17655	chr13:102875652	missense	C:G	0.939	99.0	0.495	254 (25.6%)	495 (49.8%)	245 (24.6%)
	rs873601	chr13:102875987	3'UTR	G:A	0.814	100.0	0.478	271 (27.0%)	506 (50.4%)	227 (22.6%)
POLE2	rs6572597	chr14:49662797	intron	A:C	0.813	100.0	0.178	677 (67.4%)	297 (29.6%)	30 (3.0%)
MNAT1	rs17256107	chr14:60762073	intron	A:G	0.708	100.0	0.277	527 (52.5%)	397 (39.5%)	80 (8.0%)
	rs4151190	chr14:60786235	intron	A:G	0.652	100.0	0.317	465 (46.3%)	442 (44.0%)	97 (9.7%)
	rs2300471	chr14:60797351	intron	A:G	0.081	100.0	0.124	765 (76.2%)	230 (22.9%)	9 (0.9%)
	rs2020892	chr14:60811966	intron	A:G	1.000	99.9	0.108	798 (79.6%)	193 (19.2%)	12 (1.2%)
	rs4151240	chr14:60829515	intron	G:A	0.047	100.0	0.149	719 (71.6%)	271 (27.0%)	14 (1.4%)
	rs4151279	chr14:60869987	intron	A:C	1.000	99.6	0.067	870 (87.0%)	126 (12.6%)	4 (0.4%)
	rs4151285	chr14:60870717	intron	A:T	1.000	99.2	0.130	755 (75.8%)	224 (22.5%)	17 (1.7%)
	rs4151351	chr14:60916029	intron	G:A	0.159	99.8	0.411	336 (33.5%)	508 (50.7%)	158 (15.8%)
	rs4151361	chr14:60931688	intron	A:C	0.803	99.9	0.120	775 (77.3%)	215 (21.4%)	13 (1.3%)
	rs4151374	chr14:60935235	intron	A:G	0.609	100.0	0.325	453 (45.1%)	449 (44.7%)	102 (10.2%)
	rs7156087	chr14:60935600	intron	G:A	0.204	99.9	0.197	640 (63.8%)	331 (33.0%)	32 (3.2%)
	rs4151393	chr14:60952744	intron	G:A	1.000	99.9	0.124	770 (76.8%)	218 (21.7%)	15 (1.5%)
	rs4151405	chr14:60967503	intron	A:C	0.211	100.0	0.170	685 (68.2%)	296 (29.5%)	23 (2.3%)

	rs4151408	chr14:60968059	intron	G:A	0.770	99.9	0.171	691 (68.9%)	281 (28.0%)	31 (3.1%)
ERCC4	rs3136038	chr16:13919522	5'flanking	G:A	1.000	100.0	0.253	559 (55.7%)	381 (37.9%)	64 (6.4%)
	rs31870	chr16:13943873	intron	G:A	0.975	100.0	0.193	654 (65.1%)	312 (31.1%)	38 (3.8%)
	rs1799801	chr16:13948101	synonymous	A:G	0.508	99.9	0.233	586 (58.4%)	367 (36.6%)	50 (5.0%)
	rs11075223	chr16:13950860	3'UTR	C:A	0.760	100.0	0.226	599 (59.7%)	356 (35.5%)	49 (4.9%)
	rs4781562	chr16:13951379	3'UTR	C:A	0.624	100.0	0.231	591 (58.9%)	363 (36.2%)	50 (5.0%)
RPA1	rs12952857	chr17:1829006	5'flanking	A:G	1.000	100.0	0.453	300 (29.9%)	498 (49.6%)	206 (20.5%)
	rs8065937	chr17:1829683	5'flanking	C:A	0.593	100.0	0.397	369 (36.8%)	472 (47.0%)	163 (16.2%)
	rs5030749	chr17:1830105	synonymous	A:G	0.970	100.0	0.369	401 (39.9%)	466 (46.4%)	137 (13.6%)
	rs2287321	chr17:1853057	intron	G:A	0.951	100.0	0.440	316 (31.5%)	493 (49.1%)	195 (19.4%)
	rs6416887	chr17:1854184	intron	A:G	0.995	99.6	0.197	646 (64.6%)	315 (31.5%)	39 (3.9%)
	rs11078676	chr17:1865283	intron	G:A	0.409	100.0	0.250	560 (55.8%)	387 (38.5%)	57 (5.7%)
	rs3786136	chr17:1869979	intron	G:A	1.000	100.0	0.143	737 (73.4%)	246 (24.5%)	21 (2.1%)
	rs12150513	chr17:1871298	intron	A:C	0.370	98.9	0.235	576 (58.0%)	368 (37.1%)	49 (4.9%)
	rs8067195	chr17:1874978	intron	A:G	0.363	99.9	0.171	693 (69.1%)	276 (27.5%)	34 (3.4%)
	rs2230930	chr17:1879663	synonymous	G:A	0.902	100.0	0.449	303 (30.2%)	500 (49.8%)	201 (20.0%)
	rs9909163	chr17:1885352	intron	A:G	1.000	100.0	0.378	388 (38.6%)	473 (47.1%)	143 (14.2%)
	rs2270412	chr17:1888880	intron	G:A	0.150	100.0	0.323	450 (44.8%)	460 (45.8%)	94 (9.4%)
	rs2230931	chr17:1891886	synonymous	A:G	0.527	100.0	0.267	535 (53.3%)	402 (40.0%)	67 (6.7%)
	rs12051574	chr17:1896306	intron	G:A	0.051	100.0	0.196	659 (65.6%)	296 (29.5%)	49 (4.9%)
	rs17339284	chr17:1896364	intron	G:A	0.709	100.0	0.213	619 (61.7%)	342 (34.1%)	43 (4.3%)
	rs7406953	chr17:1896662	intron	A:G	0.044	100.0	0.193	664 (66.1%)	292 (29.1%)	48 (4.8%)
	rs4790838	chr17:1896793	intron	A:G	0.139	100.0	0.358	403 (40.1%)	484 (48.2%)	117 (11.7%)
	rs12727	chr17:1897771	3'UTR	C:G	0.180	99.5	0.136	751 (75.2%)	224 (22.4%)	24 (2.4%)
	rs17734	chr17:1897850	3'UTR	A:G	0.831	100.0	0.472	278 (27.7%)	505 (50.3%)	221 (22.0%)

ERCC2	rs13181	chr19:45351661	missense	A:C	0.904	100.0	0.083	843 (84.0%)	155 (15.4%)	6 (0.6%)
	rs1052555	chr19:45352266	synonymous	G:A	1.000	100.0	0.064	879 (87.5%)	121 (12.1%)	4 (0.4%)
	rs3916874	chr19:45353668	intron	G:C	0.632	99.8	0.181	669 (66.8%)	303 (30.2%)	30 (3.0%)
	rs238416	chr19:45353791	intron	G:A	0.685	100.0	0.470	286 (28.5%)	493 (49.1%)	225 (22.4%)
	rs50871	chr19:45359257	intron	A:C	0.960	99.9	0.275	526 (52.4%)	402 (40.1%)	75 (7.5%)
	rs1799793	chr19:45364001	missense	G:A	0.240	98.7	0.059	880 (88.8%)	105 (10.6%)	6 (0.6%)
	rs238406	chr19:45365051	synonymous	C:A	0.656	99.4	0.466	280 (28.1%)	505 (50.6%)	213 (21.3%)
	rs3810366	chr19:45370684	5'flanking	C:G	0.402	99.9	0.497	247 (24.6%)	516 (51.4%)	240 (23.9%)
ERCC1	rs3212986	chr19:45409478	3'UTR	C:A	0.104	100.0	0.336	455 (45.3%)	424 (42.2%)	125 (12.5%)
	rs3212965	chr19:45417322	intron	G:A	0.077	99.9	0.324	471 (47.0%)	414 (41.3%)	118 (11.8%)
	rs3212964	chr19:45417538	intron	G:A	0.892	98.7	0.423	331 (33.4%)	481 (48.5%)	179 (18.1%)
	rs3212961	chr19:45419065	intron	C:A	0.573	99.9	0.462	295 (29.4%)	489 (48.8%)	219 (21.8%)
	rs3212955	chr19:45420238	intron	A:G	0.398	99.1	0.312	477 (47.9%)	415 (41.7%)	103 (10.4%)
	rs11615	chr19:45420395	synonymous	G:A	0.418	100.0	0.234	594 (59.2%)	350 (34.9%)	60 (6.0%)
LIG1	rs274883	chr19:48119288	intron	A:G	0.429	100.0	0.289	502 (50.0%)	424 (42.2%)	78 (7.8%)
	rs251690	chr19:48121469	intron	A:G	0.802	100.0	0.418	342 (34.1%)	484 (48.2%)	178 (17.7%)
	rs2288881	chr19:48136655	intron	G:A	0.727	100.0	0.161	708 (70.5%)	268 (26.7%)	28 (2.8%)
	rs3730931	chr19:48143984	intron	A:G	0.925	100.0	0.127	765 (76.2%)	222 (22.1%)	17 (1.7%)
	rs20580	chr19:48151296	synonymous	A:C	0.949	100.0	0.291	505 (50.3%)	413 (41.1%)	86 (8.6%)
	rs274861	chr19:48156481	intron	G:A	0.503	100.0	0.126	764 (76.1%)	227 (22.6%)	13 (1.3%)
	rs4801749	chr19:48164034	intron	A:C	1.000	99.9	0.128	763 (76.1%)	224 (22.3%)	16 (1.6%)
	rs20579	chr19:48165573	5'UTR	G:A	1.000	99.8	0.127	763 (76.1%)	223 (22.3%)	16 (1.6%)
POLD1	rs3219281	chr19:50383830	5'flanking	G:A	0.909	100.0	0.141	739 (73.6%)	246 (24.5%)	19 (1.9%)
	rs3219305	chr19:50387351	intron	C:G	0.745	98.5	0.209	616 (62.3%)	332 (33.6%)	41 (4.1%)

rs2546551	chr19:50393103	intron	G:A	1.000	100.0	0.153	720 (71.7%)	261 (26.0%)	23 (2.3%)
rs3219341	chr19:50394760	intron	G:A	0.942	100.0	0.142	739 (73.6%)	244 (24.3%)	21 (2.1%)
rs1726801	chr19:50401817	missense	G:A	0.780	99.0	0.140	736 (74.0%)	237 (23.8%)	21 (2.1%)
rs1673041	chr19:50406132	intron	A:C	0.799	99.9	0.301	492 (49.1%)	418 (41.7%)	93 (9.3%)

Supplementary Table1 173 SNPs information

[a] SNP position in GRCh38.p7.

Patient characteristic		Gastrointestinal Toxicity		Hematological Toxicities					
				Neutropenia		Anemia		Thrombocytopenia	
		No./Total[c]	P^d	No./Total[c]	P^d	No./Total[c]	P^d	No./Total[c]	P^d
Gender			**1.4×10^{-6}**		0.654		0.309		0.056
	Male	37/674		79/659		18/666		19/670	
	Female	43/290		36/276		11/278		15/280	
Age			0.472		0.260		0.455		0.891
	≤58	38/495		54/485		17/489		18/492	
	＞58	42/469		61/450		12/455		16/458	
ECOG PS			0.197		0.674		0.099		1.000
	0-1	70/869		101/843		24/852		31/858	
	2	10/82		11/81		5/81		3/81	
Smoking Status			**1.5×10^{-4}**		0.820		**0.042**		0.447
	Non-smoker[a]	49/408		47/393		17/395		16/398	
	Ever smoker	29/552		67/538		11/545		17/548	
TNM Stage			0.583		0.283		**0.020**		0.927
	IIIA	6/80		6/76		0/81		3/81	
	IIIB	20/283		30/270		4/275		9/275	
	IV	54/596		79/584		25/583		22/589	
Histological Type			0.650		0.978		0.368		0.499
	Adenocarcinoma	52/609		74/593		23/598		25/601	
	Squamous Cell Carcinoma	14/213		24/206		3/208		5/209	
	Adenosquamocarcinoma	2/19		2/20		0/19		1/20	
	Others[b]	12/123		15/116		3/119		3/120	

Chemotherapy Regimens		0.091		**3.4×10^{-4}**		0.086		**5.8×10^{-6}**
Platinum-navelbine	30/293		54/284		13/292		7/295	
Platinum-gemcitabine	21/235		15/232		7/232		20/233	
Platinum-paclitaxel	17/305		33/294		6/295		2/295	
Platinum-docetaxel	5/84		10/80		0/81		1/82	
Others platinum combinations	7/47		3/45		3/44		4/45	

Supplementary Table 2 Covariants for Toxicities

Abbreviations: ECOG PS, Eastern Cooperative Oncology Group performance status; No., numbers; TNM, tumor-node metastasis.

[a] Non-smokers were defined as those who had smoked <1 cigarette per day and for <1 year in their lifetime.

[b] Other carcinomas included mixed cell or undifferentiated carcinoma.

[c] Numbers indicate the patients who experienced grade 3 or 4 toxicities.

[d] $P<0.05$ was shown in blod.

Patient characteristic		Response Rate		Clinical Benefit[c]		OS		PFS	
		No./Total[d]	P^{e}	No./Total[d]	P^{e}	MST	Log-rank P^{e}	MST	Log-rank P^{e}
Gender			0.280		0.161		**0.002**		0.660
	Male	131/689		564/689		18.1		7.7	
	Female	46/286		223/286		22.5		9.5	
Age			0.176		0.897		**0.003**		0.340
	≤58	83/502		406/502		21.3		9.5	
	＞58	94/473		381/473		17.2		7.7	
ECOG PS			0.686		**0.010**		0.100		**0.001**
	0-1	162/878		718/878		19.4		9.3	
	2	14/84		59/84		17.9		5.4	
Smoking Status[a]			0.338		0.351		**0.014**		0.540
	Non-smoker	69/411		326/411		21.2		7.8	
	Ever smoker	108/563		460/563		17.9		9.5	
TNM Stage			**2.1×10^{-4}**		0.138		0.132		0.122
	IIIA	27/79		70/79		23.0		12.6	
	IIIB	56/284		230/284		19.1		9.8	
	IV	94/607		481/607		19.1		8.0	
Histological Type			**1.7×10^{-7}**		**0.006**		0.057		0.850
	Adenocarcinoma	78/612		477/612		20.2		9.1	
	Squamous Cell Carcinoma	62/217		186/217		16.6		9.5	
	Adenosquamocarcinoma	3/19		13/19		15.3		12.5	
	Others[b]	34/127		111/127		18.0		7.6	

Chemotherapy Regimens		**0.025**		0.507		0.305		**4.0×10^{-6}**
Platinum-navelbine	73/302		250/302		19.9		13.8	
Platinum-gemcitabine	37/231		188/231		19.8		9.3	
Platinum-paclitaxel	49/310		248/310		18.3		6.6	
Platinum-docetaxel	11/85		67/85		17.7		7.2	
Others platinum combinations	7/47		34/47		20.2		5.1	

Supplementary table 3 Covariants for Response and Survival

Abbreviations: ECOG PS, Eastern Cooperative Oncology Group performance status; MST, median survival time (months); No., numbers; OS, overall survival (months); PFS, progression-free survival (months); TNM, tumor-node metastasis;

[a.] Non-smokers were defined as those who had smoked <1 cigarette per day and for <1 year in their lifetime.

[b.] Other carcinomas included mixed cell or undifferentiated carcinoma.

[c.] Clinical benefit was defined as patients with response of complete response (CR), partial response (PR) or stable disease (SD).

[d.] Numbers indicate the patients who clinical benefit in the same genotype group.

[e.] *P*<0.05 was shown in blod; Data in italics means Fisher's Exact Test was used.
